# Antenatal diagnosis of hydrometrocolpos with Mullerian duplication on ultrasound and fetal MRI: case report and literature review

**DOI:** 10.1259/bjrcr.20230024

**Published:** 2023-05-22

**Authors:** Abhinav Chander Bhagat, Radha Sarawagi Gupta, Rajesh Malik

**Affiliations:** 1 All India Institute of Medical Sciences, Bhopal, India

## Abstract

Fetal abdomino-pelvic cystic lesions are uncommon and can have varied etio-pathogenesis. Most commonly they originate from the gastrointestinal or genitourinary tract. These include choledochal cyst, hydronephrosis, renal cyst, mesenteric/omental cyst, ovarian cyst, meconium pseudocyst, and hydrocolpos/hydrometrocolpos among others. Fetal hydrometrocolpos is rare with a reported incidence of 0.006% and its diagnosis requires a high index of suspicion. Antenatal ultrasound and magnetic resonance imaging (MRI) is invaluable in diagnostic evaluation. This case report describes the imaging features of antenatally detected congenital hydrometrocolpos with Mullerian duplication secondary to cloacal malformation using antenatal ultrasound and MRI. Per-operative findings and other possible differential diagnoses are discussed along with a brief review of literature.

## Introduction

Fetal abdomino-pelvic cystic lesions are uncommon and can have varied etio-pathogenesis. Most commonly they originate from the gastrointestinal or genitourinary tract. These include choledochal cyst, hydronephrosis, renal cyst, mesenteric/omental cyst, ovarian cyst, meconium pseudocyst and hydrocolpos/hydrometrocolpos among others.^1^ Most of these lesions are incidentally detected during routine antenatal ultrasound examination. Fetal hydrometrocolpos is rare with a reported incidence of 0.006%, diagnosis of which requires a high index of suspicion.^2^ Careful scrutiny of the morphology of lesion, its location, and relationship with normal structures aids in suggesting the appropriate differential diagnoses, and in some cases may even reveal the exact diagnosis. Fetal magnetic resonance imaging (MRI) is invaluable in confirming the ultrasound findings and improving the diagnostic accuracy.^3^

## Case report

### Antenatal history

A 27-year-old primigravida with non-consanguineous marriage presented to our institution for the first time at 30 weeks of gestation. Anomaly scan done at an outside institution at 21 weeks reported a distended urinary bladder with upstream hydronephrosis. The patient’s antepartum history was normal. There was no significant previous medical and surgical history. She was referred for routine ultrasound evaluation. Maternal aneuploidy markers were normal.

### Antenatal ultrasound findings

**Figure 1.**
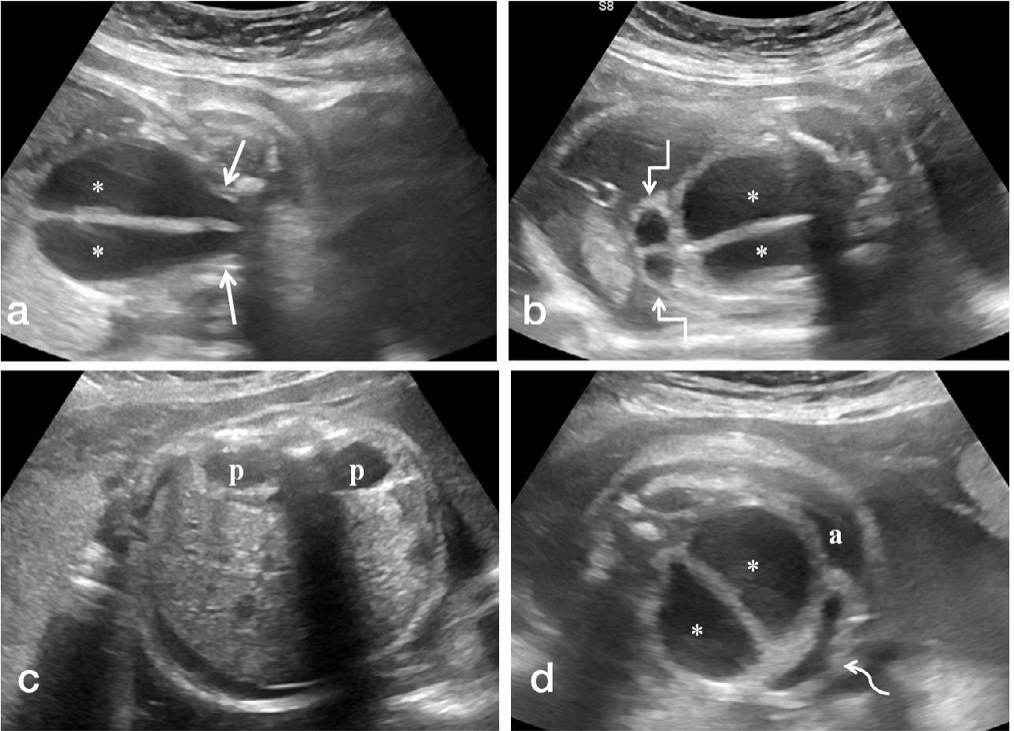
(a-d). Antenatal ultrasound images in coronal plane (a,b) showing the distended vagina (*) with central echogenic vertical septum funneling towards the pelvis (straight arrows) and distended hemiuteri at the cranial aspect of distended vagina (square arrows). Images in the axial plane (c,d) reveal bilateral renal pelvic dilatation (p) and anteriorly displaced urinary bladder (curved arrow) with fetal ascites (a).

Trans-abdominal ultrasound scan was performed using GE LogiqS8 ultrasound machine. A single live intrauterine pregnancy was discovered. The placenta was anterior and not low-lying with adequate amniotic fluid index. Two-vessel umbilical cord was seen. Upon scanning the fetal abdomen, there was a large central elongated midline cystic lesion of size 3.9 × 4.5 cm with a 5-mm-thick midline septum and fine internal echoes (Figure 1). This cyst was seen to funnel inferiorly into the pelvis and interpreted as two hemivaginas. Fetal bladder was visualized and seen displaced anteriorly by the mass against anterior abdominal wall. There was single umbilical artery surrounding the bladder. At the superior aspect of this mass, two small fluid-filled structures measuring 1 × 1 cm showing thick walls were identified which were thought to represent two hemiuteri. Bilateral kidneys were identified and showed dilated renal pelvis with anteroposterior pelvic diameter (APPD) ranging between 1.3 and 1.5 cm. There was associated renal cortical thinning with increased cortical echogenicity. Fetal perianal muscle was not identified despite careful scrutiny. The scrotum was not visualized and an elongated phallus like structure (measuring 1.8 cm in length) with labia was seen. Mild ascites was also present. No other structural anomalies were identified. Based on this constellation of findings, a presumptive diagnosis of congenital hydrometrocolpos with longitudinal vaginal septum and uterus didelphys was suggested. Fetal growth parameters were normal except for increased abdominal circumference owing to abdominal distension due to the cystic mass and ascites. Doppler ultrasound parameters were normal. The patient was followed-up after 2 weeks and repeat scanning revealed mild increase in the size of the cyst as well as APPD.

### Antenatal MRI findings

**Figure 2.**
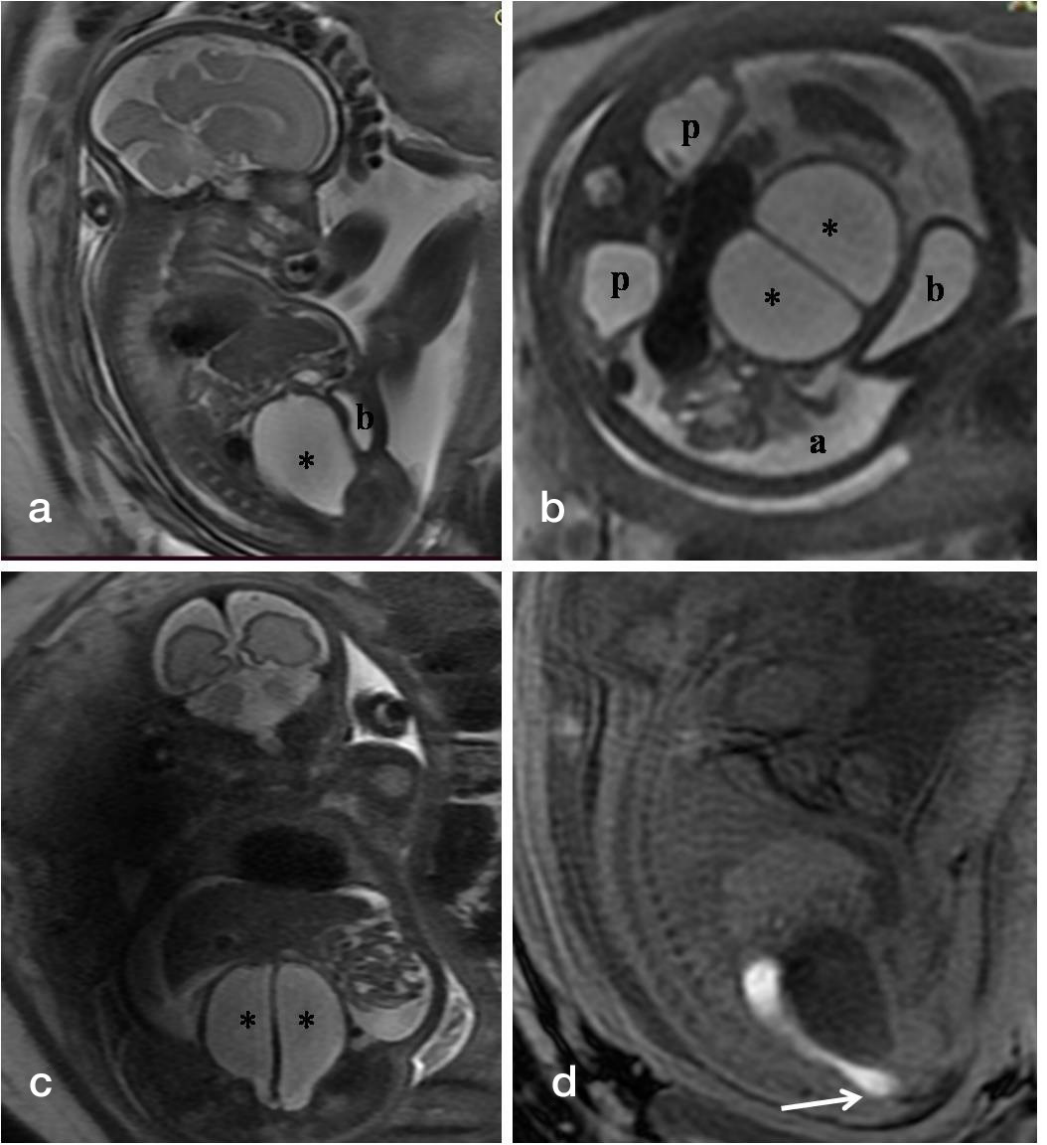
(a-d). T_2_-weighted fetal MR images in sagittal (a), axial (b), and coronal (c) planes show the distended vagina (*) with central linear hypointense vertical septum displacing the bladder (b) anteriorly against the abdominal wall, bilateral renal pelvic dilatation (p) and fetal ascites (a). Sagittal T_1_-weighted MR image (d) reveals hyperintense meconium within non-dilated rectum seen reaching upto the perineum.

Fetal MRI was performed on 3 Tesla GE Discovery MR750w machine on the same day as initial ultrasound scan. Sagittal *T*_1_-weighted images along with sagittal, coronal, and axial *T*_2_-weighted images of the fetus were acquired. A T1 hypointense and T2 hyperintense cystic mass was identified between the bladder and rectum with a vertical T2 hypointense midline septum (Figure 2). There were two similar but smaller fluid-filled structures present at the cranial end of the cyst representing mild dilatation of the endometrial cavity of the two hemiuteri. Sagittal *T*_1_-weighted images revealed hyperintense meconium in the rectum which was extending towards the perineum. Bilateral renal pelvic dilatation was also present secondary to compression by the cystic mass. Apart from mild fetal ascites, rest of the study was normal. These findings corroborated the ultrasound study and were congruous with the diagnosis of congenital hydrometrocolpos with uterus didelphys, possibly secondary to urogenital sinus. Cloacal malformation was considered less likely due to the presence of meconium in the rectum extending upto the perineum and the absence of colonic dilatation.

### Labor and delivery outcome

The patient was admitted in ward and put under observation, while the neonatology and pediatric surgery teams were informed. The baby was delivered at 35 weeks 4 days via normal vaginal delivery which was uneventful. The neonate was a female weighing 1.97 kg with spontaneous cry at birth. The renal function was deranged with uncompensated metabolic acidosis for which sodium bicarbonate injection was given and subsequently renal function recovered. No other complications were present apart from abdominal distension due to the antenatally diagnosed cystic mass. Apgar scores of 9 and 10 were noted at 1 and 5 min, respectively. The baby was admitted to the pediatric surgery neonatal intensive care unit (ICU) for further investigations.

### Postnatal care and per-operative findings

The neonate was passing urine and meconium through a single opening. Physical examination revealed clitoral hypertrophy (2 cm) and single opening in the vestibule with absent anal and vaginal opening. Upon inserting the feeding tube through the opening in vestibule, there was a common channel of length 5 mm, beyond which two openings were identified: anterior opening was common for urethra and vagina and a posterior opening for rectum. Karyotyping revealed 46 XX type. No other systemic abnormalities were detected upon screening for VACTERL association.

At one week of age, the baby underwent exploratory laparotomy. Per-operative findings revealed dilated and hypertrophied bladder reaching till the umbilicus. Posterior to the bladder, dilated vagina was present which had a vertical septum with two hemi-uteri on top of it. There was pus discharge from left fallopian tube with multiple flimsy adhesions between the bowel loops. Thus, the final diagnosis was cloacal malformation with longitudinal vaginal septum and uterus didelphys. Division of the vaginal septum was done, tube vaginostomy was performed and a catheter was placed in the right hemivagina. Transverse divided colostomy and surface vesicostomy was also performed. The baby was discharged in a stable condition and definitive surgery was planned in the subsequent follow-up visits.

## Discussion

Fetal hydrocolpos is a rather rare condition having an estimated incidence of 0.006% .^2^ Most frequently, it is discovered as an incidental abdomino-pelvic cystic lesion during routine antenatal ultrasound evaluation in late second or third trimester.^4^ Owing to secretion of fluid by secretory glands in the cervix and vagina under the influence of maternal hormones, these get collected in the obstructed vagina which then gets distended and presents as a cystic mass; alternately vaginal distension can occur due to collection of urine due to presence of urogenital sinus or cloacal malformation.^5^

Obstructive causes of hydrocolpos which impede the drainage of vaginal secretions include imperforate hymen, distal vaginal agenesis, and transverse vaginal septum. The most common cause has been stated to be imperforate hymen.^6^ However, urogenital sinus and cloacal malformation may also result in hydrocolpos.

During embryogenesis, the cloaca appears as the terminal part of the hindgut and forms a common recess for the urogenital and anorectal tract in the fifth week. The urorectal septum extends caudally and partitions the cloaca into two separate channels (urogenital sinus and rectum) during the sixth and seventh weeks. Upon contact with the inferiorly growing urorectal septum, the cloacal membrane ruptures resulting in the creation of two orifices. The urogenital sinus undergoes further differentiation and forms the urinary bladder and urethra, along with a part of the vagina in females. Cloacal malformation ensues if the urorectal septum fails to join the cloacal membrane during this period.^7^ Consequently, there is a confluence of the lower urinary tract, the female reproductive tract, and the rectum into a common channel which exteriorizes via a single perineal opening. In urogenital sinus, the urethra and vagina form a common channel of variable length which opens through a single perineal orifice along with a separate opening for the anus.^8^

Cloacal abnormalities can present as a spectrum of malformations, including cloacal dysgenesis, classic cloaca, posterior cloaca, urogenital sinus, cloacal variant and posterior cloacal variant, each with its own characteristic features.^9^ Cloacal malformations have been divided into two clinical groups based on the length of the common channel, which dictates surgical management. A common channel less than 3 cm in length is associated with a lower incidence of other related abnormalities and a comparatively simpler surgical reconstruction. On the other hand, a common channel greater than 3 cm in length is linked with a higher incidence of associated anomalies, difficult surgical repair, and poorer functional results.^10^ Another classification categorizes cloacal malformation into three subtypes depending on the length of common channel and urethra: (a) Type 1 (common channel length<1 cm), (b) Type 2 (common channel length<3 cm and urethral length atleast 1.5 cm); and (c) Type 3 cloaca (common channel length>3 cm or urethra<1.5 cm).^11^

In around 40% of patients with cloacal malformation, there is associated Mullerian duplication comprising two hemiuteri and two hemivaginas separated by an intervening septum. This septum can be partial/total and symmetric/asymmetric. Two major complications may stem from hydrocolpos: (a) it may exert compression on the trigone of bladder, producing vesico-ureteric reflux, megaureter, and hydronephrosis; and (b) the hydrocolpos may get secondarily infected, if left undrained, resulting in pyocolpos. Reflux of fluid/secretions into the peritoneal cavity by way of fallopian tubes can cause fetal ascites and later on lead to development of endometriosis, pelvic adhesions, and infertility.^12,13^

There are many characteristic ultrasound imaging features which can help in diagnosis. Hydrocolpos is seen as a midline elongated pear-shaped retrovesical pelvic/abdomino-pelvic cystic mass, often displacing and compressing the bladder against the anterior abdominal wall. A potential source of diagnostic error is misidentification of hydrocolpos as distended bladder. Recurrent distension and emptying at different scan times helps confirm that it is urinary bladder; additionally, identification of umbilical arteries on its either side is also helpful in identifying the bladder. Hydrocolpos, on the other hand, has an elongated shape and shows funneling inferiorly toward the pelvis. At the superior aspect of the hydrocolpos, one may visualize the imprint of cervix and identify the uterus, which itself may contain fluid, although the presence of uterine musculature results in far lesser uterine distension as compared to vagina. Furthermore, the presence of a midline septum within the cyst goes in favor of hydrocolpos. These features, along with urinary tract dilatation due to mass effect and fetal ascites secondary to escape of urine and secretions into the peritoneal cavity by way of fallopian tubes, are highly suggestive of underlying cloacal malformation.

In our case, based on antenatal ultrasound and MRI, possibility of urogenital sinus was favored over classic cloaca due to visualization of meconium-filled rectum on *T*_1_-weighted images extending into the perineum and the absence of rectal dilatation. However, it turned out to be classic cloacal malformation. In classic cloaca, one would expect a high position of the recto-sigmoid cul-de-sac due to agenesis of the distal bowel segment, along with dilated colon. We posit that the extremely short length of common channel (5 mm) in our case as confirmed on post-natal examination may be responsible for the visualization of meconium-filled non-dilated rectum extending into the perineum. Presumably, with increase in the length of common channel, the typical findings of classic cloaca *i.e*. absent rectal meconium and colonic dilatation will predominate.

There are several other findings that may be present in association with cloacal malformations such as oligohydramnios (in patients with severe urinary obstruction and renal dysplasia), polyhydramnios [in cases with esophageal atresia with VACTERL (vertebral defects, anal atresia, cardiac defects, tracheoesophageal fistula, renal anomalies, and limb abnormalities) association], lumbo-sacral spinal abnormalities (tethered cord, hypoplastic sacrum, sacral hemivertebrae), non-visualization of the anus and ambiguous genitalia.^14^ Hydrometrocolpos may also be seen in association with such syndromes as McKusick Kaufman syndrome (hydrometrocolpos, congenital heart disease, and post-axial polydactyly), Bardet–Biedl syndrome (hydrometrocolpos, rod-cone dystrophy, post-axial polydactyly, learning difficulties, obesity, and renal abnormalities), Mayer–Rokitansky–Küster–Hauser syndrome [congenital aplasia of the uterus and upper vagina, renal (unilateral agenesis, horseshoe kidney, ectopic kidneys), skeletal (Klippel-Feil anomaly), hearing defects, cardiac and digital anomalies (syndactyly, polydactyly)] and OHVIRA (obstructed hemivagina and ipsilateral renal anomaly)/Herlyn–Werner–Wunderlich syndrome.^15^

Other differentials of cystic abdominal masses to be considered include ovarian cyst, intestinal duplication cyst, meconium cyst, etc.; however, imaging features such as location on either side of urinary bladder, presence of gut signature, identification of echogenic calcified wall, respectively, can help in differentiation with these entities.

The management of cloacal anomalies is primarily surgical. Initial surgery aims to decompress the vagina, perform urinary diversion, and create a temporary colostomy. This is followed by definitive surgical repair (depending on the length of common channel) to reconstruct the anatomy, with the aim to achieve bowel and urinary continence along with normal sexual function.^12^

## Conclusion

Congenital hydrometrocolpos with Mullerian duplication is a rare anomaly. Radiologist should be familiar with the characteristic ultrasound imaging findings as they can be the first to suggest the diagnosis of an underlying cloacal malformation. With the help of a careful ultrasound evaluation, diagnosis can be made with good certainty. Fetal MRI helps provide detailed assessment of pelvic anatomy rendering it valuable in confirmation of diagnosis and as a problem-solving tool. Early diagnosis with prompt referral and management in dedicated neonatology/pediatric surgery neonatal ICU is essential to prevent complications and ensure good quality of life for the patient.

## Learning points

- Congenital hydrometrocolpos with Mullerian duplication is a rare entity and should be included in the differential diagnosis of antenatal abdomino-pelvic cystic mass.
- It is important not to mistake distended vagina for urinary bladder.
- Ultrasound and MR imaging features are fairly characteristic to allow an accurate diagnosis, so radiologists should be familiar with the imaging features so as to initiate appropriate post-natal management.
